# Knowledge, Attitude, and Practices (KAP) of Human Populations towards Malaria Control in Four Ecoepidemiological Settings in Cameroon

**DOI:** 10.1155/2021/9925135

**Published:** 2021-06-11

**Authors:** Nelly Armanda Kala Chouakeu, Laura Gilberine Ngingahi, Roland Bamou, Abdou Talipouo, Carmen Sandra Ngadjeu, Marie Paul Audrey Mayi, Edmond Kopya, Parfait Awono-Ambene, Timoléon Tchuinkam, Christophe Antonio Nkondjio

**Affiliations:** ^1^Vector-Borne Diseases Laboratory of the Research Unit of Biology and Applied Ecology (VBID-RUBAE), Department of Animal Biology, Faculty of Science, University of Dschang, Dschang, Cameroon; ^2^Organisation de Coordination pour la lutte contre les Endémies en Afrique Centrale (OCEAC), Yaoundé, Cameroon; ^3^Laboratory of Parasitology and Ecology, Faculty of Sciences, University of Yaoundé, Yaoundé, Cameroon

## Abstract

Malaria remains a major public health problem in Cameroon. Critical steps to improve disease control include assessing human population adherence to vector control interventions, especially in areas with different cultural backgrounds. The present study seeks to assess the knowledge, attitude, and practices (KAP) of populations towards malaria prevention in four ecoepidemiological settings in Cameroon. A cross-sectional malaria KAP survey was conducted from August to September 2019 in households of the localities of Kaélé, Tibati, Bertoua, and Santchou. A semistructured questionnaire was administered to randomly selected households in the localities. Data recorded were analysed using SPSS v. 20 and MedCalc v14.8.1. A total of 739 households were surveyed. The majority of participants had a secondary level of education (48.71%). A substantial number of participants (over 90%) in all the localities were able to make an accurate association between malaria and mosquito bites. The main sources of information for community members were television sets in Santchou and Tibati and interpersonal conversations in Bertoua and Kaélé. Mosquito nets were the most commonly used protective measure against malaria, and the majority of nets in households came from the free-of-charge mass distribution campaigns organized by the government. Participants with secondary and higher levels of education were more aware of good practices towards malaria control compared to those with a primary level of education. The study revealed that populations' KAP differed according to localities and culture. More sensitization and education need to be done to improve adherence to prevention programs.

## 1. Introduction

Malaria still contributes intensely to the global health burden [1]. The disease is also considered a barrier to the socioeconomic development of sub-Saharan countries [2]. According to recent statistics, half of the world's population lives in malaria-endemic settings and about 229 million people suffer from malaria attacks yearly, with 409,000 associated annual deaths [1]. Sub-Saharan Africa remains the most affected region with more than 93% (213 million) of malaria cases and 94% of deaths, which further contribute to the impoverishment of the region [1]. In Cameroon, malaria is a major public health concern and the entire country is exposed to the risk of transmission [3]. According to the Ministry of Public Health, in 2018, 2,133,523 malaria cases were recorded in health facilities, representing 25.9% of outpatient consultations and 3,299 deaths. About 65% of deaths due to malaria were children under 5 years [3].

The fight against malaria remains a priority for many countries in sub-Saharan Africa. In the absence of an effective vaccine, disease prevention relies almost exclusively on vector control interventions. In Cameroon, vector control mainly relies on the mass distribution of insecticide-treated nets (ITNs) [4–6]. Between 2000 and 2016, the frequent distribution of ITNs across the country and improvements in the diagnosis and management of malaria cases using artemisinin-based combination therapy resulted in a decrease in malaria incidence from 41% in 2013 to 24.3% in 2016 [7]. In addition, mortality in healthcare facilities decreased from 25% in 2013 to 14.6% in 2018 [5]. However, despite the increased scale-up of ITNs, residual malaria transmission remains a challenge in most epidemiological settings with high malaria incidence [8–10]. Several factors could be responsible for the subperformance of current control interventions. These include the rapid expansion of insecticide resistance, which is largely expanding in the country, changes in vector resting feeding and biting behaviour, and the poor usage of treated nets by the population [6, 11–16].

To improve the usage of bed nets by the community and the sustainability of vector control interventions, a good understanding of local population beliefs, knowledge, and practices in relation to disease transmission and prevention is required [17, 18]. Studies conducted elsewhere showed that population adherence to any control programme and their willingness to accept interventions were influenced by their cultural background [19].

Since 2005, several studies have been conducted to assess the knowledge, attitude, and practices (KAP) of the local population on malaria prevention in Cameroon [4, 6, 20–23]. However, there are still not enough studies comparing population knowledge and practices in different ecoepidemiological settings. The present study was designed to assess the KAP towards malaria of people living in four ecoepidemiological settings with different cultural backgrounds in Cameroon. The ultimate goal of the study was to generate key information to inform policymakers in developing strategies to improve the use of control interventions in these localities.

## 2. Materials and Methods

### 2.1. Vector-Borne Diseases Characteristics and Description of the Study Sites

In Cameroon, malaria affect all the country, however, with different endemicity levels according to ecological settings varying from hyperendemic to hypoendemic settings [12]. Due to the high endemicity of malaria in the country, malaria cases in most ecological settings are mainly indigenous cases. Yet, it is possible that for urban sites such as Bertoua, which receive frequent arrival of migrants or due to frequent exchanges between regions, that part of malaria cases could be imported cases.

Other vector-borne diseases of medical importance recorded in the country include dengue, chikungunya, Zika virus, onchocerciasis, and human African trypanosomiasis. These diseases are largely distributed across the country and affect a large proportion of the population.

**Figure 1:**
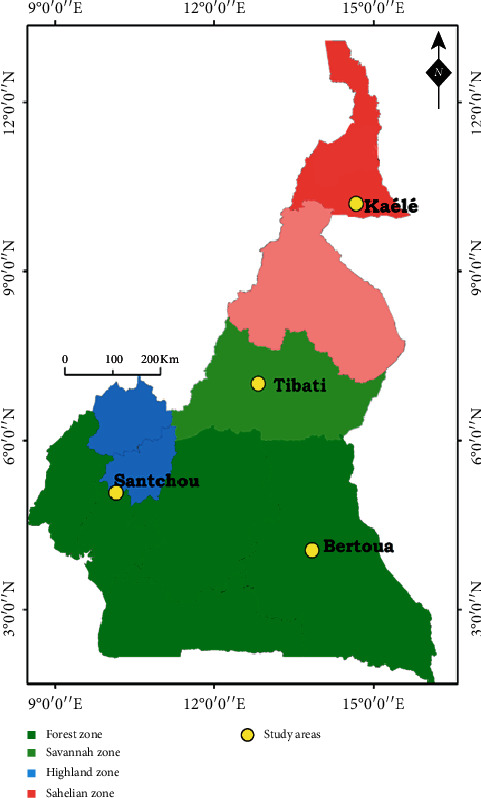
Map of the study area, Cameron.

The study was conducted in four ecoepidemiological settings in Cameroon: Kaélé in the Sahelian zone, Tibati in the Sahelo-Sudanese area, Bertoua in the forested area, and Santchou in the highland area. The description of the different sites is provided in Table 1.  Figure 1 shows the map of study areas.

### 2.2. Study Design and Data Collection

The study is a descriptive cross-sectional survey to assess population KAP on malaria prevention and treatment in Kaélé, Tibati, Bertoua, and Santchou. Data were collected using a pretested questionnaire. Interviewers were trained on how to use the questionnaire and on methods to approach respondents and obtain consent before the beginning of the survey. All the interviewers were students with at least a master's level.

The survey was conducted from August to September 2019 during the rainy season. The questionnaire was administered to randomly selected households. The interviewees were the heads of households and in their absence, a responsible adult above 18 years was interviewed. Only households where informed consent was obtained were enrolled in the study. The questionnaire was prepared in French and sometimes translated into English or communicated in local languages by a guide when necessary. Interviews were undertaken during 10–15 minutes in private to reduce the influence from other people. The questionnaire consisted of 31 questions and included two parts. The first part collected sociodemographic details (sex, occupation, level of education, number of persons living in the house, and number of children under five years). The second part included questions designed to assess the knowledge and practices of participants about malaria transmission, prevention, and treatment.

### 2.3. Data Analysis

KAP survey responses were coded then entered into a Microsoft Excel database. Data cleaning was performed to check for inconsistencies in data entry and responses and transferred to the SPSS software package (SPSS version 22, Chicago, IL) for analyses. Categorical variables were reported using descriptive statistics such as frequencies and percentages. Variables analysed included knowledge of the vector, sociodemographic factors, malaria-related KAP. Frequencies were compared using Chi-squared test. Different outcomes were also evaluated: (i) the proportion of households that own at least a net; (ii) the proportion of households that own at least one LLIN for 2 people; (iii) the proportion of the population with access to a LLIN within the household; (iv) the proportion of the population that used a LLIN the previous night. To identify factors associated with knowledge on malaria and usage of protection measures, the odds ratios (OR) and their 95% confidence intervals (95% CI) were calculated using MedCalc v14.8.1 software. Statistical significance was set at *P* < 0.05.

To assess the knowledge of respondents on malaria transmission, answers to the questions concerning how malaria is transmitted to man and the ability to identify anopheline as a vector of malaria were considered. Participants providing correct answers to these two questions were considered as having good knowledge of malaria transmission. Concerning good practices in regard to malaria prevention and treatment, three questions were assessed, including possessing ITNs, sleeping under a treated bed net regularly, and going to the hospital for malaria treatment. Participants respecting these practices were considered as applying good practices, while those with fewer than three correct answers were considered as having poor practices.

### 2.4. Ethical Approval and Consent to Participate

The study was conducted under the ethical clearance N° 2020/04/1209/CE/CNERSH/SP delivered by the Cameroon National Ethics (CNE) Committee for Research on Human Health.

## 3. Results

### 3.1. Sociodemographic Characteristics of Households Surveyed

A total of 739 households were surveyed. The number of households interviewed according to localities is presented in Table 2. In Kaélé, all respondents were men (100%). In Kaélé and Tibati, the majority of respondents had a primary school level (40.4% in Kaélé and 60% in Tibati), while in Bertoua and Santchou, the majority of respondents attended secondary school (50.41% in Bertoua and 69.5% in Santchou). In all the localities, most households heads interviewed were doing small businesses (>60%). Different house characteristics were registered during the study. The majority of houses were of traditional style constructed with mud in Kaélé (100%) and Tibati (90.82%) or with cement blocks in Bertoua (60.39%) and Santchou (82.03%). The roof included iron sheet in Bertoua (85.71%) and Santchou (93.05%) with open eaves and ceiling. In Kaélé and Tibati, most houses had thatched roofs and no ceiling. People in Kaélé kept their domestic animals inside houses during the night, whereas in the three other localities, they were kept in enclosures.

### 3.2. Malaria Knowledge: Information, Education, and Communication

Over 90% of respondents in each locality attributed the transmission of malaria to mosquito bites and indicated to be bitten every night by mosquitoes. The most frequently reported signs and symptoms of malaria in all the localities included vomiting, headaches, and fever (>80% of respondents).

### 3.3. Sources of Information about Malaria

**Figure 2:**
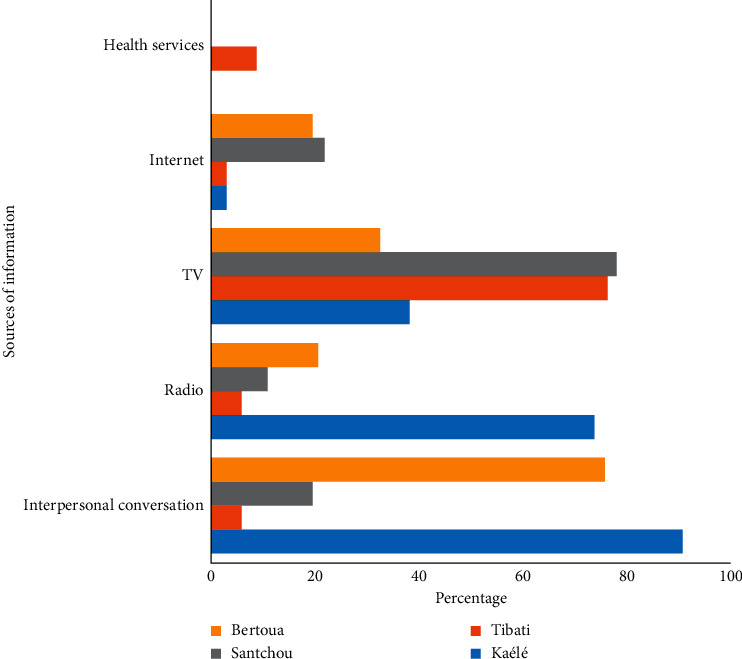
Sources of information about malaria in Kaélé, Tibati, Santchou, and Bertoua.

The main sources of information concerning malaria for the population included watching and listening to TV (Television) programs, interpersonal conversations, radio, and Internet (Figure 2). Watching TV programs was significantly high in Santchou and Tibati (*P* < 0.0001), while listening to the radio was the main source of information in Kaélé (*P* < 0.0001). Interpersonal conversations were mostly used in Kaélé (90.90%) and Bertoua (76.08%) compared to Santchou. Internet was also used in Bertoua and Santchou as a source of information. Just a few people reported getting information from the hospital.

### 3.4. Knowledge on the Mode of Transmission of Malaria and Use of Preventive Measures

Over 90% of respondents in each locality attributed the transmission of malaria to mosquito bites and indicated to be bitten every night by mosquitoes (Table 3).

The main tool used to prevent mosquito bites in the four sites was ITNs, followed by insecticide sprays with as active ingredient pyrethroids (deltamethrin, permethrin, and alpha-cypermethrin). In Kaélé, people reported using mosquito nets exclusively, while in the three remaining sites, people were using both treated nets and insecticide sprays. Ranking choices concerning periods when treated nets were used, the majority of people reported regularly using ITNs in Kaélé (98.1%) and Tibati (90%), while just about 50% of people in Bertoua and Santchou reported using them regularly. Some participants (about 30%) declared using treated nets during the rainy season. The majority of bed nets available in households in Kaélé (96.84%) and Santchou (94.11%) are derived from mass distribution campaigns. About 29% of people interviewed in Tibati and Bertoua reported having bought their bed nets in the market. Most of the bed nets inspected were more than two years old (Table 3).

### 3.5. Ownership and Usage of Insecticide-Treated Nets in Households

The proportion of households owning at least a net varied from 60.68% in Bertoua to 93.91% in Kaélé. The proportion of households possessing a LLIN for two people was 50% in Kaélé, Bertoua, and Santchou, while it was only 16% in Tibati. The proportion of the population who had access to a LLIN within their household varied from 43% to 76.04%, depending on the locality. The proportion of the population that used a LLIN the previous night was 34.43% in Tibati, 41.11% in Bertoua, 72.70% in Santchou, and 76.04% in Kaélé (Table 4).

### 3.6. Care-Seeking Attitude and Financial Cost of Malaria Treatment

In Kaélé, Bertoua, and Santchou, over 80% of the heads of households reported going to the hospital when they suspect a malaria case. Plants (traditional medicine) were also used for the treatment of malaria particularly in Tibati (83.33%). Despite the fact that the majority of the respondents reported visiting the hospital in case of malaria, they also practiced self-medication (Table 5).

Regarding the treatment of malaria cases, the average amount spent for malaria treatment per year varied from 14000 to 26000 FCFA (28 to 52 US dollars) (Table 5).

### 3.7. Association between Sex, Level of Education, Profession, and Knowledge of Malaria Transmission

Comparisons were conducted to assess any association between good knowledge of malaria vectors and different factors related to the respondents in the four localities. In Bertoua, men had better knowledge of the mode of transmission of malaria compared to women (OR = 1.82; *P*=0.04). In addition, participants with secondary and university levels of education had good knowledge of malaria transmission compared to illiterate participants (OR = 6.91, *P*=0.0002; OR = 10.59, *P* < 0.001 respectively). Civil servants had good knowledge compared to housewives and those doing small-scale business (Table 6).

### 3.8. Association between Sex, Level of Education, Profession, and Usage of Prevention Tools

Comparisons were also made to assess any association between good practices concerning malaria prevention and treatment and different factors related to the respondents in the four localities. In Santchou, men were less aware of good practices concerning malaria prevention and treatment compared to women (OR = 0.50; *P*=0.01). Participants with secondary and higher levels of education were more aware of good practices compared to those with primary level. Civil servants were more aware of good practices compared to housewives and those doing small-scale business (Table 7).

None of the factors were significantly associated with practices in Kaélé and Tibati.

## 4. Discussion

One of the most important ways for improving malaria vector control is to understand factors affecting the adherence of communities to vector control interventions. The objective of the study was to assess the knowledge and practices of communities living in four ecoepidemiological settings in Cameroon with different cultural backgrounds.

A substantial number of respondents in the four localities were able to make an accurate association between malaria and mosquito bites. This good knowledge could be attributed to the high endemicity of malaria in the four localities, the increased communication on the disease over radio, TV, and newspapers, and different sensitization campaigns conducted by the NMCP [28, 29].

The main sources of information for the population were TV programmes in Santchou and Tibati and interpersonal conversations in Bertoua and Kaélé. Health facilities were also used by some individuals in Tibati to fetch information. These data are in agreement with previous studies which also identified different audiovisual platforms such as television, radio, newspapers, and Internet (Facebook, WhatsApp, and Twitter) as a source of information for local communities [19, 30]. Concerning the use of interpersonal conversation in Bertoua and Kaélé, it is likely that the frequent shortage of electric power in these cities at the time of the study may have promoted this communication means. Studies in South Africa and Zambia indicated that health facilities could also serve as a primary source of information on malaria [8, 31]; however, just few participants went to hospitals to look for information.

The majority of participants in the four study sites (Kaélé, Bertoua, Tibati, and Santchou) were able to identify the most common malaria signs and symptoms (fever, headache, and vomiting). This result matches the WHO target for knowledge [32] and is in line with findings from other studies [19, 31, 33].

Pyrethroid-treated mosquito nets were the most commonly used protective measure in all localities, followed by insecticides (deltamethrin/permethrin) spray/coils, and netting at doors or windows in Santchou, Bertoua, and Tibati. Our findings are consistent with the findings from previous studies in Cameroon [4, 6, 34].

Over 71% (71–96.84%) of people interviewed in the four study sites declared using regularly impregnated bed nets to prevent malaria. Similar results were obtained by previous studies in Yaoundé, Douala, Bafang, and Bamenda [4, 6, 20, 21]. This high usage rate might be linked to the high ownership derived from the free distribution campaigns of LLINs to the population carried out by the NMCP [7]. Different net brands have been distributed to the population [35]. The usage rate recorded during the present study was far higher than estimates in the general population; this difference could come from the fact that the usage rate was assessed through self-reports. It is likely that usage rate might be less important than data recorded and could be due to the fact that participants responded regularly using nets because they thought that was the best answer awaited from them.

Up to 50% of people interviewed in Tibati said they had nets partly or completely damaged, which could be explained by the fact that the last distribution campaign took place in 2015, five years before the study. Mosquito nets degradation 5 years after distribution could be the result of frequent or bad utilization of this tool by household members or the poor quality of the material used. However, because an ITN could be effective for at least 3 years under field conditions, WHO recommends that large-scale distribution campaigns should be conducted every 3 years [36].

The proportion of households possessing at least a net was high in the four study sites. The proportion of households possessing one bed net for two people was, however, significantly low and was consistent with previous findings in the city of Yaoundé [6]. It is, however, frequent to find a high number of people sleeping in a room, some using and some not using nets. This situation could increase the exposure of non-net users to mosquito bites.

Several factors were found to contribute to the low usage rate of nets, including feeling heat when sleeping under a net and forgetting to put on the mosquito net before sleeping. This situation requests further sensitization of the population to improve good practices. It was also noted that some people used treated nets in agriculture for the protection of young plants (Bertoua and Santchou) and for fishing and farming (Kaélé and Tibati). These poor practices have been highlighted in previous studies [6, 20, 37] and request further attention.

Over 50% of people interviewed in Kaélé, Bertoua, and Santchou reported going to the hospital when they suspect a case of malaria, maybe because malaria treatment is free of charge for children in Cameroon [3]. Yet, important variations were recorded between sites. In Tibati, certain participants also reported preferring using traditional medicine or consulting traditional healers. This suggests a strong attachment to their traditional culture.

The amount spent annually by families for malaria prevention and treatment was largely above estimates that were reported in previous studies [38]. These observations are consistent with the high malaria endemicity in these localities.

Participants with secondary and higher levels of education were more aware of good practices compared to those with a primary level of education. Civil servants were more aware of good practices compared to housewives and those doing small-scale business. These are consistent with studies conducted in Cameroon and elsewhere [6, 39]. Indeed, educated people are more likely to be reached by malaria messages on different audiovisual platforms such as television, radio, newspapers, Internet, while this is not the case for less educated people.

## 5. Conclusion

Knowledge, attitude, and practices survey in four ecoepidemiological settings revealed significant differences in practices concerning malaria prevention and treatment in the study sites. This information should be used for the development of new approaches to improve communities' adherence to vector control interventions. Knowledge was out of step with practices in most of the localities. It now becomes important that education programs mainly address best practices to improve the adherence of local communities to vector control programs.

## Figures and Tables

**Table 1:** Description of the study sites.

Characteristics	Kaélé	Tibati	Bertoua	Santchou
Region name	Far north	Adamawa	East	West
Coordinates	10–50'36”N; 14–56'23”E	12–37'60'N; 12–37'60”E	4–34–30“N; 13–41–4”E	5–58'27”N; 9–58' 27'E
Domain	Sahelian zone	Sahelo-Sudanese (humid savannah)	Forest zone	Highland grassfields
Urban/rural	Rural	Rural	Urban	Rural
Altitude	340 m above sea	840 m above sea level	400 m above sea level	750 m above sea level
Climate	Sahelian	Tropical humid	Subtropical	Equatorial
Average temperature	33°C	28°C	26°C	23°C
Seasons	A long dry season (9 months) and a short rainy season	A dry and rainy season of similar length (6 months each)	4 seasons: two rainy and two dry	A long rainy season (8 months) and a short dry season (4 months)
Vegetation	Wooded Savanah	Grassy Savanah	Semi‐deciduous dense forest	Grassland
Main ethnic groups	Guiziga, Peulh, Moundang, Toupouri, Mafa	Peulhs, Tikar, Mboum, Gbaya, Haoussa	Beti, Hausa, Baka, Bamileké	Mbô, Bamileké, and Bamoun
Main activities	Commercial, fishing, farming	Cattle breeding, commercial	Civil servants, small business	Agriculture, small business
Religion	>90% Muslims	70% Muslims, 30% Christian	30% Muslim, 70% Christian	>80% Christian
Endemic stratum	Seasonal hyperendemic	Mesoendemic	Mesoendemic	Hypoendemic
Entomological inoculation rate	2.4–24.0 infected bites/person/month [24]	100 infected bites/person/year [25].	20–50 infected bites/person/year [12]	0.03–2.24 infected bites/person/month [26, 27]
Main malaria vectors	*An*. *arabiensis, An*. *gambiae*, *An*. *funestus*	*An*. *gambiae*, *An*. *coluzzii*	*An*. *gambiae*, *An*. *coluzzii*	

**Table 2:** Sociodemographic characteristics of households surveyed in Kaélé, Tibati, Santchou, and Bertoua.

Categories	Characteristics	Kaélé	Tibati	Bertoua	Santchou
		N (%)	N (%)	N (%)	N (%)
*Gender*	Male	99 (100)	76 (66.1)	196 (74.8)	160 (60.8)
	Female	0	39 (33.9)	66 (25.2)	103 (39.2)

*Education level*	Primary level	40 (40.41)	69 (60)	45 (18.29)	43 (16.60)
	Secondary level	32 (32.32)	23 (20)	124 (50.41)	180 (69, 5)
	University level	00 (00)	00 (00)	58 (23.58)	25 (9.65)
	None	27 (27.27)	23 (20)	19 (7.72)	11 (4.24)

*Average number of people per home*	All people	5.97 (2–25)	6.14 (1–15)	4.49 (1–14)	5.97 (1–20)
	Children <5	2.15 (1–6)	2.80 (1–8)	0.82 (1–4)	1.09 (1–5)

*Occupation of head of household*	Small business	92 (93.88)	91 (81.98)	103 (61.67)	195 (75.88)
	Civil servant	4 (4.08)	0 (0)	36 (21.55)	52 (20.23)
	Housewife	0 (0.00)	19 (17.11)	20 (11.98)	6 (2.33)
	Student	1 (1.02)	1 (0.90)	85 (50.90)	4 (1.55)

*House construction material*	Cement blocks	0 (0)	6 (5.71)	154 (60.39)	210 (82.03)
	Mud and cement	0 (0)	0 (00)	54 (21.17)	34 (13.28)
	Clay	99 (100)	99 (94.29)	13 (5.10)	12 (4.69)
	Plank	00 (00)	00 (00)	34 (13.33)	00 (00)

*Roof*	Tiles/iron sheet	41 (41.83)	38 (34.23)	216 (85.71)	241 (93.05)
	Thatched	57 (58.16)	73 (65.77)	27 (10.71)	17 (6.56)
	Other	00 (00)	00 (00)	9 (3.57)	1 (0.38)

*Eaves*	Present	00 (00)	46 (44.23)	68 (32.23)	100 (39.37)
	Absent	100 (00)	58 (55.77)	143 (67.77)	154 (60.63)

*Ceiling*	Present	3 (3.03)	7 (6.54)	156 (60.23)	129 (53.75)
	Absent	96 (96.97)	100 (93.46)	103 (39.77)	111 (46.25)

*Domestic animals*	Present	83 (84.69)	62 (55.36)	44 (17.25)	80 (34.04)
	Absent	15 (15.31)	50 (44.64)	211 (82.74)	155 (65.95)

*Resting places for domestic animals*	Inside house	83 (100)	28 (41.79)	3 (7.14)	6 (7.32)
	Same room	00 (00)	4 (5.97)	13 (30.95)	12 (14.63)
	Other	00 (00)	00 (00)	2 (4.76)	2 (2.44)
	Enclosures	00 (00)	35 (52.24)	24 (57.14)	62 (75.61)

*Water source*	Tap water	00 (00)	0 (00)	58 (22, 13)	30 (11, 41)
	Well	21 (21, 20)	69 (60)	75 (28, 62)	125 (47.53)
	Natural source	00 (00)	6 (5.22)	88 (33.59)	32 (12.17)
	Drilling water	77 (77.80)	81 (70.43)	169 (64.50)	84 (31.94)
	Mineral water	0 (00)	2 (1.74)	13 (4.96)	3 (1.14)

Percentages do not add up to 100 because these results are from multiple response questions; N: number.

**Table 3:** Population knowledge and attitude concerning malaria prevention and usage of LLINs in Kaélé, Tibati, Santchou, and Bertoua.

Variables	Answers	Kaélé	Tibati	Bertoua	Santchou
		N (%)	N (%)	N (%)	N (%)
*Mode of transmission of malaria*	Mosquito bites	93 (94)	83 (76.15)	174 (84.1)	229 (90.87)
	Dirt	0	1 (0.92)	00 (00)	19 (7.54)
	Cold	0	6 (5.50)	1 (0.4)	1 (0.4)
	Do not know	6 (6)	19 (17.43)	32 (15.31)	3 (1.1)

*Mosquito bites at night*	yes	99 (100)	115 (100)	247 (96.48)	242 (92.01)

*Prevention measures used*	Mosquito nets	99 (100)	102 (88.69)	245 (93.51)	253 (96.19)
	Insecticides sprays/coils	0 (00)	21 (18.26)	55 (20.99)	91 (34.60)
	Screen nets on windows	0 (00)	4 (3.48)	6 (2.29)	6 (2.28)

*Period of use of Mosquito nets*	Rainy season	1 (1.90)	5 (5.00)	82 (33.74)	76 (29.69)
	Dry season	0 (00)	5 (5.00)	26 (10.70)	5 (1.95)
	Regularly	98 (98.10)	90 (90)	135 (55.55)	176 (68.75)

*Origin of bed nets used*	Freely acquired	92 (96.84)	68 (71)	125 (71.84)	240 (94.11)
	Bought	3 (3.15)	28 (29)	49 (28.16)	15 (5.89)

*Age of bed nets used*	<6 months	2 (2.10)	3 (3.15)	79 (33.76)	158 (61.71)
	>6 months	00 (00)	7 (7.37)	78 (33.33)	37 (14.45)
	>1 years	2 (2.10)	10 (10.53)	31 (13.24)	32 (12.5)
	>2 years	91 (95.8)	75 (78.95)	46 (19.65)	29 (11.33)

*Physical integrity of mosquito nets*	Good	60 (62.22)	50 (45.45)	210 (85.71)	173 (67.05)
	Damaged	38 (38.78)	60 (54.55)	35 (14.29)	85 (32.95)

*Reasons for not using mosquito nets regularly*	Absence of mosquito nets	0 (0)	69 (84.15)	22 (11.96)	21 (33.87)
	Heat	1 (100)	8 (9.75)	65 (35.33)	36 (58.06)
	Forgetting	0 (0)	5 (6.10)	97 (52.72)	5 (8.06)

N: total of respondents.

**Table 4:** Ownership and usage of insecticide-treated nets in households in Kaélé, Tibati, Santchou, and Bertoua.

Sites	% HHs owning ≥1 LLIN	% HHs owning ≥1 LLIN for 2 people	% population with access to a LLIN within their own HH	% population that used a LLIN the previous night	Ratio usage: access
Kaélé	80.80	57.57	76.04	76.04	1
Tibati	68.69	16.52	43	34.43	0.80
Bertoua	60.68	50.38	47.3	41.11	0.87
Santchou	93.91	59.31	73.94	72.70	0.98

HHs: households; LLIN: insecticide-treated nets, ratio = usage/access.

**Table 5:** Home management of malaria cases and financial cost of malaria treatment in Kaélé, Tibati, Santchou, and Bertoua.

Items	Characteristics	Kaélé	Tibati	Bertoua	Santchou
		N (%)	N (%)	N (%)	N (%)
*Management of malaria cases*	Hospital	99 (100)	60 (62.5)	95 (90.48)	89 (65.44)
	Self-medication	0	28 (29.17)	44 (41.9)	43 (31.62)
	Traditional medicine	0	80 (83.33)	15 (14.29)	18 (13.23)

*Expenses*	For malaria treatment	26172.83 ± 10121.5	14000 ± 7375.63	16230.76 ± 15880.56	17540 ± 12751.04

Percentages do not add up to 100 because these results are from multiple response questions; N: number.

**Table 6:** Factors associated with good knowledge about malaria in the study sites.

	Localities	Kaélé			Tibati			Bertoua			Santchou		
	Variables	N	GK (%)	OR (95% CI)	N	GK (%)	OR (95% CI)	N	GK (%)	OR (95% CI)	N	GK (%)	OR (95% CI)
*Gender*	Female	0	—	—	39	32	1	66	37	1	103	91	1
	Male	99	—	—	76	51	0.45 (0.17–1.15)	196	137	1.82 (1.02–3.23)^*∗*^	160	138	0.83 (0.39–1.75)

*Level of education*	Illiterate	27	25	1	21	17	1	20	9	1			
	Primary	39	37	1.48 (0.19–11.21)	69	45	0.44 (0.13–1.46)	45	21	1.07 (0.37–3.08)	43	37	1
	Secondary	32	30	1.2 (0.16–9.14)	23	19	1.12 (0.24–5.17)	124	81	6.91 (3.48–13.81)^*∗∗∗*^	180	158	1.16 (0.44–3.07)
	Higher	0	0	—	0	0		58	52	10.59 (3.12–35.9)^*∗∗∗*^	25	23	1.86 (0.35–10.03)

*Occupation*	Housewives	1	1	1	20	17	1	37	31	3.23 (1.24–10.00)^*∗∗*^	52	48	1
	Civil servant	4	3	0.78 (0.02–32.37)	—	—	—	103	63	1.35 (0.6–3.05)	196	167	0.48 (0.16–1.43)
	Small business	93	88	5.36 (0.19–147.55)	91	62	0.37 (0.10–1.39)	21	10	1	7	6	0.50 (0.05–5.24)

N: total; GK: good knowledge; OR: odds ratio; ^*∗*^*P* ≤ 0.05; ^*∗∗*^*P* ≤ 0.001; ^*∗∗∗*^*P* ≤ 0.0001.

**Table 7:** Factors associated with good practices about malaria in the study sites.

	Localities	Kaélé			Tibati			Bertoua			Santchou		
	Variables	N	GP (%)	OR (95% CI)	N	GP (%)	OR (95% CI)	N	GP (%)	OR (95% CI)	N	GP (%)	OR (95% CI)
*Gender*	Female	0	—	—	39	34	1	66	39	1	103	78	1
	Male	99	—	—	76	56	0.41 (0.14–1.2)	196	96	0.66 (0.38–1.17)	160	98	0.5 (0.29–0.88)^*∗*^

*Level of education*	Illiterate	27	27	1	21	17	1	20	13	1			
	Primary	39	38	0.47 (0.02–11.89)	69	56	1.01 (0.29–3.52)	45	24	0.61 (0.21–1.83)	43	22	1
	Secondary	32	32	1.18 (0.02–61.55)	23	16	0.54 (0.13–2.19)	124	59	0.49 (0.18–1.31)	180	128	2.35 (1.19–4.63)^*∗*^
	Higher	—	—	—	—	—	—	58	34	0.76 (0.26–2.19)	25	16	0.16 (0.30–0.85)^*∗*^

*Occupation*	Housewives	1	1	1	20	19	1	37	28	4.13 (1.35–12.67)^*∗*^	52	43	1
	Civil servant	4	4	3 (0.04–228.68)	—	—	—	103	45	1.30 (0.52–3.24)	196	123	0.35 (0.16–1.43)^*∗∗*^
	Small business	83	82	18.33 (0.51–660.95)	91	68	0.15 (0.02–1.23)	21	9	1	8	6	0.02 (0.06–0.05)^*∗∗∗*^

N: total; OR: odds ratio; GP: good practices; ^*∗*^*P* ≤ 0.05; ^*∗∗*^*P* ≤ 0.001; ^*∗∗∗*^*P* ≤ 0.0001.

## Data Availability

The datasets supporting the findings are included within this article.

## Abbreviations

ITNs: Insecticide-treated nets
LLINs: Long-lasting insecticidal nets
IRS: Indoor residual spraying
WHO: World Health Organization
MoH: Ministry of Public Health
NMCP: National Malaria Control Program
OR: Odds ratio
CI: Confidence interval
SPSS: Statistical Package for the Social Sciences
GK: Good knowledge
GP: Good practices.
